# A High-Throughput Biosensing Approach for Rapid Screening of Compounds Targeting the hNav1.1 Channel: Marine Toxins as a Case Study

**DOI:** 10.3390/md23030119

**Published:** 2025-03-09

**Authors:** Huijing Shen, Yuxia Cui, Shiyuan Liang, Shuang Zhou, Yingji Li, Yongning Wu, Junxian Song

**Affiliations:** 1 School of Food and Health, Beijing Technology and Business University, Beijing 100048, China; 2250021020@st.btbu.edu.cn (H.S.); wuyongning@cfsa.net.cn (Y.W.); 2 NHC Key Laboratory of Food Safety Risk Assessment, Food Safety Research Unit (2019RU014) of Chinese Academy of Medical Science, China National Center for Food Safety Risk Assessment, Beijing 100021, China; liangshiyuan@cfsa.net.cn (S.L.); szhoupku@gmail.com (S.Z.); 3 Department of Cardiology, Center for Cardiovascular Translational Research, Beijing Key Laboratory of Early Prediction and Intervention of Acute Myocardial Infarction, Peking University People’s Hospital, Beijing 100044, China; yuxia87320@126.com; 4 ICE Bioscience Inc., Beijing 100176, China; liy@ice-biosci.com

**Keywords:** voltage-gated sodium channels, hNav1.1, fluorescence sensing detection, high-throughput screening, marine toxins

## Abstract

Voltage-gated sodium (Nav) channels play a crucial role in initiating and propagating action potentials throughout the heart, muscles and nervous systems, making them targets for a number of drugs and toxins. While patch-clamp electrophysiology is considered the gold standard for measuring ion channel activity, its labor-intensive and time-consuming nature highlights the need for fast screening strategies to facilitate a preliminary selection of potential drugs or hazards. In this study, a high-throughput and cost-effective biosensing method was developed to rapidly identify specific agonists and inhibitors targeting the human Nav1.1 (hNav1.1) channel. It combines a red fluorescent dye sensitive to transmembrane potentials with CHO cells stably expressing the hNav1.1 α-subunit (hNav1.1-CHO). In the initial screening mode, the tested compounds were mixed with pre-equilibrated hNav1.1-CHO cells and dye to detect potential agonist effects via fluorescence enhancement. In cases where no fluorescence enhancement was observed, the addition of a known agonist veratridine allowed the indication of inhibitor candidates by fluorescence reduction, relative to the veratridine control without test compounds. Potential agonists or inhibitors identified in the initial screening were further evaluated by measuring concentration–response curves to determine EC_50_/IC_50_ values, providing semi-quantitative estimates of their binding strength to hNav1.1. This robust, high-throughput biosensing assay was validated through comparisons with the patch-clamp results and tested with 12 marine toxins, yielding consistent results. It holds promise as a low-cost, rapid, and long-term stable approach for drug discovery and non-target screening of neurotoxins.

## 1. Introduction

Voltage-gated sodium (Nav) channels are transmembrane proteins expressed in excitable cells that initiate and propagate action potentials among the heart, muscle, nervous system or other tissues of animals [1,2]. Human Nav channels are associated with disorders such as chronic pain, epilepsy, and cardiac arrhythmia and thus serve as targets for many drugs [3], with well-characterized therapeutic mechanisms and binding sites. Until now, nine subtypes—Nav1.1~Nav1.9 and Nav2.1—have been isolated and identified in humans [4], comprising a pore-forming α-subunit associated with auxiliary β-subunits. The α-subunit consists of four homologous repetitive functional regions (I~IV), each containing six transmembrane helices (S1~S6), with S5 and S6 from all four repeats constituting the ion transport pore and S1~S4 forming a voltage-sensing domain (VSD) in each repeat [5]. Among the nine subtypes, Nav1.1 is primarily expressed in the human central nervous system, where pathological loss of function leads to brain disorders, such as epilepsy, Alzheimer’s disease, and autism [6].

The binding of compounds towards Nav channel can be broadly classified into two categories, blocking the channel pore to inhibit the sodium conductance and altering the channel gating by interfering with the VSDs [5]. Some small-molecule neurotoxins such as tetrodotoxin (TTX) function as pore blockers, whereas most peptidic toxins are gating modifiers interacting with one or more VSDs to inhibit [7] or agonize [8] the channel [9]. Pore blockers are typically nonselective neurotoxins, whereas gating modifiers exhibit greater selectivity and hold potential to be developed as subtype-selective Nav channel drugs [10]. Currently, at least eight binding sites have been identified on Nav channels, each distinguished by its specific localization and action effects, as detailed in several comprehensive reviews [11,12].

Since the early stages of research on the mechanism of Nav channel functioning, marine toxins fulfilled an important job [11]. Marine toxins are specialized metabolic components of marine organisms, which are mainly produced by phytoplankton or microorganisms [13]. Most of them are highly toxic, accumulating and enriching in particular species through the food chain [14]. Seafood is a significant source of marine toxins for humans, and incidents of poisoning resulting from the consumption of seafood occur from time to time. Among many marine toxins, the most toxic and lethal ones are neurotoxins, including tetrodotoxin (TTX) [15], saxitoxin (STX) [16], brevetoxins [17], ciguatoxins [18], palytoxin [19] and their structural analogs [20,21,22]. They can act specifically on a variety of Nav channels, serving as either pore blockers or gating modifiers upon specific binding, thereby affecting action potentials and excitatory signaling between nerve and muscle cells [23] and causing severe acute toxic symptoms in the nervous, respiratory, and cardiovascular systems, even leading to death [24].

Patch-clamp electrophysiology has been considered the gold standard for measuring ion channel behavior, since it allows sufficient voltage control and yields millisecond-scale temporal resolution on detailed mechanistic information [25,26,27]. However, it is laborious and has a slow throughput, limiting large-scale screening of potent drugs or toxins targeting on ion channels. The subsequently developed automated and multichannel patch-clamp techniques greatly enhance efficiency but remain costly and require specific equipment [25,28]. Robust and cost-effective screening techniques, including membrane potential assays using voltage-sensitive fluorescent dyes, have also been developed to facilitate Nav channel binding research. These studies mainly focused on screening subtype-selective inhibitors of Nav1.5 and Nav1.7 channels for treating arrhythmias or pain management [10,26,29]. However, no relevant studies focusing on Nav1.1 were reported.

The successful performance of these screening approaches relies on the quality of the ion channel expression system. Up to now, the existing methods have predominantly involved using human embryonic kidney (HEK) cells for heterologous expression of Nav channels, which is mainly attributed to their relative ease of transfection and rapid protein expression [30,31,32]. However, HEK cells express various ion channels, including voltage-gated Na^+^ channels, Ca^2+^ channels, K^+^ channels, Cl^−^ channels, etc. [33], and possess a higher baseline level of endogenous ionic activity that might complicate the interpretation of some signals in flux and membrane potential assays [34]. Chinese Hamster Ovary (CHO) cells offer several advantages over HEK cells for the heterologous production of ion channels due to their superior expression stability, scalability, and most importantly, their lower levels of endogenous ionic currents. These factors highlight CHO cells as the preferred heterologous expression system for studying Nav channel functions and their interaction with potential toxic or therapeutic compounds.

**Figure 1:**
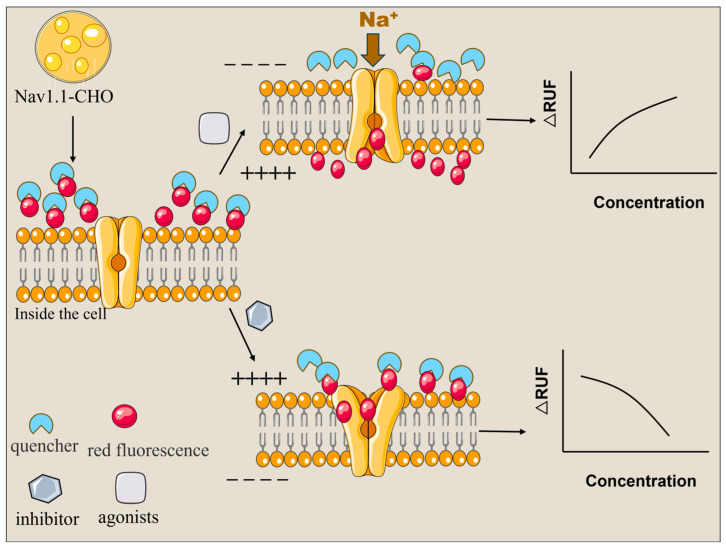
Schematic illustration of fluorescent biosensing approach for the rapid screening of agonists/inhibitors targeting hNav1.1.

In this study, based on CHO cells stably expressing hNav1.1 channels, we developed a high-throughput fluorescent biosensing approach for the rapid screening of compounds targeting hNav1.1. The principal scheme is illustrated in Figure 1. A commercially available long-wavelength fluorescent dye and its quencher were selected as indicators to swiftly and sensitively detect membrane potential changes caused by the target binding (activation or inhibition) towards the ion channels. The assay enables rapid screening of a large library of compounds in a 96-well format by observing the fluorescence enhancement or decrease. Potential agonists or inhibitors were subsequently evaluated at gradient concentrations to obtain EC_50_/IC_50_ values, achieving a semi-quantitative estimation of their binding affinities towards hNav1.1. The method was further validated using 12 marine toxins, yielding expected results comparable to patch-clamp experiments. The high-throughput, cost-effective, rapid and long-term stable assay presents an ideal alternative for drug discovery and non-target screening of neurotoxins.

## 2. Results

### 2.1. Optimization of Concentration of Veratridine in Inhibition Mode

Veratridine, a site-2 neurotoxin [35], is considered an agonist of sodium channels due to its ability to shift the activation voltage of sodium channels during depolarization and increase the probability of channel opening [36]. Its action follows a “foot-in-the-door” mechanism, whereby veratridine binds to the inner vestibule of the pore domain [37], causing persistent channel opening by hindering inactivation and deactivation [36,38].

**Figure 2:**
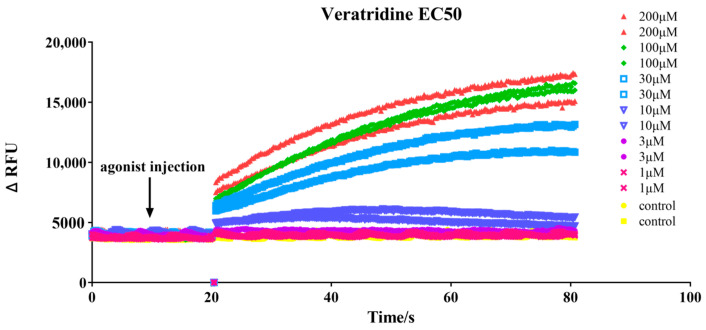
Real-time fluorescence for veratridine at various concentrations (0 μM, 1 μM, 3 μM, 10 μM, 30 μM, 100 μM, and 200 μM) on membrane depolarization in hNav1.1-CHO cells, with each concentration tested in duplicate.

In the inhibition mode, veratridine is added after the test compounds to assess their inhibitory effects on sodium channel opening. Therefore, its concentration needs to be optimized. Real-time fluorescence changes induced by veratridine across a concentration range of 0–200 μM are shown in Figure 2. As the concentration of veratridine increased, the fluorescence intensity rose accordingly, reaching a plateau at 100 μM. A concentration of 30 μM, corresponding to approximately 80% of the maximum fluorescence signal, was selected as the optimal working concentration. At this level, the fluorescence signal was sufficiently strong and could exhibit sharp changes, making it ideal for indicating the inhibitory effects.

### 2.2. Feasibility of Method

**Figure 3:**
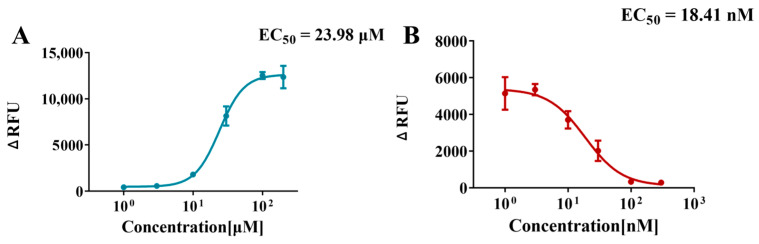
Concentration–response curves for veratridine (EC_50_ = 24 μM) (**A**) and tetrodotoxin (IC_50_ = 18.41 nM) (**B**). Data points represent the mean ± SE from triplicate measurements in three wells.

Firstly, the feasibility of the method in both agonist and inhibitor modes was confirmed using a well-known hNav1.1 channel agonist (veratridine) and inhibitor (tetrodotoxin, TTX), respectively, which served as positive controls for subsequent compound testing. As shown in Figure 2 and Figure 3A, the fluorescence intensity increased significantly as the veratridine concentration was gradually raised from 0 μM to 200 μM. The EC_50_ value for veratridine was obtained from the concentration–response curve to be approximately 24 μM, verifying the feasibility of the method in agonist mode.

The inhibitor mode was verified by TTX, a potent neurotoxin that binds to the extracellular vestibule of the sodium channel and, thus, a classic sodium channel inhibitor [39,40]. The inhibition curve for TTX, shown in Figure 3B, demonstrates that the ability of veratridine to open the sodium channel is progressively inhibited at increasing TTX concentrations. The higher the TTX concentration, the more pronounced the inhibition, as evidenced by a marked decrease in fluorescence intensity. At a TTX concentration of 100 nM, the channel-opening effect of 30 μM veratridine was almost completely inhibited. From the inhibition curve, an IC_50_ value of 18.41 nM for TTX was obtained, confirming the feasibility of the method in inhibition mode.

### 2.3. Application to Marine Toxins—Initial Screening

Nav channels serve as molecular targets for a variety of potent toxins that bind to distinct sites on the α-subunits of sodium channels, thereby affecting ion penetration and channel gating [35]. These toxins include TTX, saxitoxin (STX), and µ-conotoxin (site 1), batrachotoxin, veratridine, aconitine, and grayanotoxin (site 2), polypeptide sea anemone and α-scorpion toxins (site 3), β-scorpion toxins (site 4), brevetoxins and ciguatoxins (site 5), and δ-conotoxins (site 6) [35]. In this study, 12 marine toxins, including gonyautoxin 1/4 (GTX1/4), GTX 2/3, GTX 5, GTX 6, decarbamoylgonyautoxin 2&3 (dcGTX 2&3), STX, decarbamoylsaxitoxin (dcSTX), N-sulfocarbamoylgonyautoxin 2 and 3 (C1&C2), dinophysistoxin 1 (DTX 1), bevetoxin1, brevetoxin2, and brevetoxin3, were employed to validate the application of the established method.

**Figure 4:**
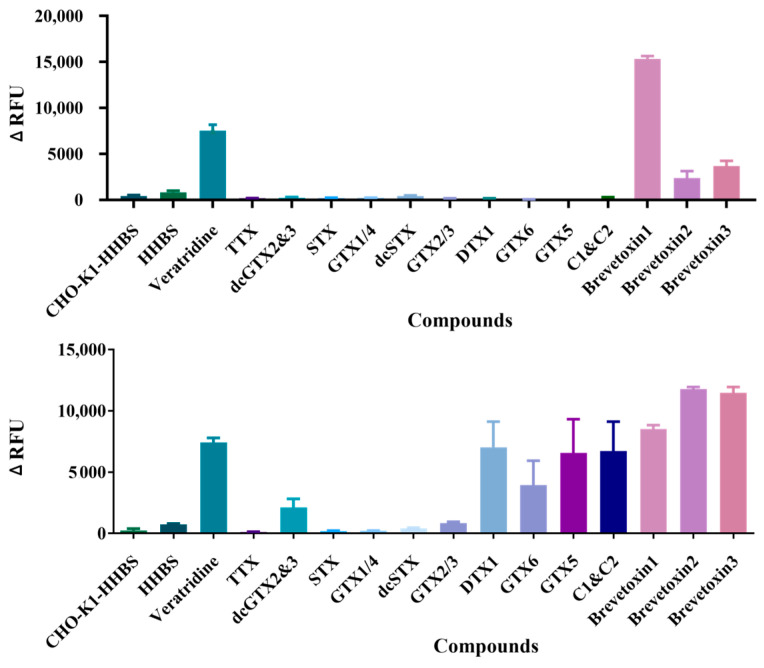
Initial screening of activation (**A**) and inhibitory (**B**) effects of 12 marine toxins on hNav1.1, including dcGTX2&3, STX, GTX1/4, dcSTX, GTX2/3, DTX1, GTX6, GTX5, C1&C2, Brevetoxin1, Brevetoxin2, and Brevetoxin3. The agonist veratridine (positive control in **A**) and inhibitor TTX (positive control in **B**) were used as quality controls. CHO-K1-HHBS serves as the cell line blank and HHBS acts as the buffer blank. The bars represent the mean ± SE from triplicate measurements.

First, the 12 marine toxins (500 nM) were tested in the initial screening mode for potential agonists. As shown in Fig. 4A, brevetoxin1, brevetoxin2, and brevetoxin3 exhibited significant agonist activity, with HHBS serving as the negative control and 30 μM veratridine as the positive control, while other toxins showed no agonistic effect. The structures of brevetoxins are characterized by a linear array of cyclic ethers with trans/syn-fused ether rings [41], a structure shared with other polycyclic ether toxins such as ciguatoxin [42]. These toxins display a variety of potent biological activities, which may contribute to their agonist properties [43]. 

In addition, the 12 marine toxins were also tested in the initial screening mode for inhibitors (100 nM test compounds + 30 μM veratridine), with 30 μM veratridine alone as the negative control and TTX as the positive control. The results, shown in Figure 4B, indicate that dcGTX2&3, STX, GTX1/4, dcSTX, and GTX2/3 exhibited strong inhibitory effects. All these toxins possess structural similarities to STX, whose binding to Nav channels is characterized by a series of key structural features, as evidenced by cryo-electron microscopy [5,44]. In contrast, GTX6 had a weak inhibitory effect, while DTX1, GTX5, and C1&C2 showed no significant inhibition at 100 nM.

When used in initial mode, the established method could primarily elucidate the binding properties of the test compounds toward the hNav1.1 channel and distinguish between their agonistic and inhibitory effects.

### 2.4. Application to Marine Toxins—Activation Curve and EC_50_ Determination

**Figure 5:**
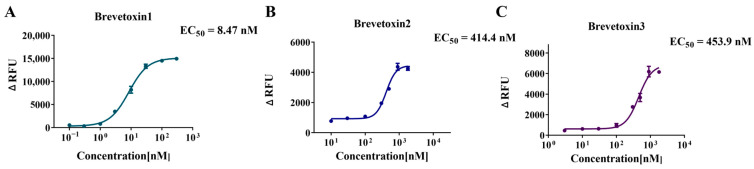
Agonist concentration–response curves and EC_50_ values of brevetoxin1 (**A**), brevetoxin2 (**B**), and brevetoxin3 (**C**). Data points represent the mean ± SE from triplicate measurements.

For the potential agonists identified in the preliminary screening, brevetoxin1, brevetoxin2, and brevetoxin3, their concentration–response curves were measured by serial dilution to further confirm their agonistic effects on hNav1.1. As shown in Figure 5, the fluorescence intensity increased significantly with the concentration of brevetoxins, further validating their agonistic properties. The EC_50_ values for brevetoxin1, brevetoxin2, and brevetoxin3 obtained from the concentration–response curves were 8.47 nM, 414.4 nM, and 453.9 nM, respectively, demonstrating that brevetoxin1 exhibited stronger agonistic activity on hNav1.1 compared to brevetoxin2 and brevetoxin3. These findings are consistent with previous studies, which have shown that brevetoxins, produced by dinoflagellates such as *Karenia brevis* during warm water red tides, can cause neurotoxic shellfish poisoning. They bind to voltage-gated sodium channels at site 5, making the channels more active by shifting the activation voltage to more negative potentials and by slowing the inactivation process [45].

### 2.5. Application to Marine Toxins—Inhibition Curve and IC_50_ Determination

**Figure 6:**
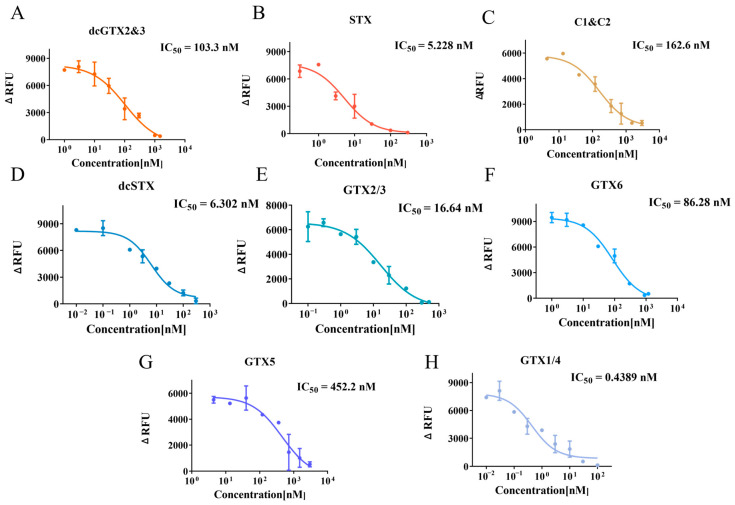
Inhibition curves and IC_50_ values for dcGTX2&3 (**A**), STX (**B**), C1&C2 (**C**), dcSTX (**D**), GTX2/3 (**E**), GTX6 (**F**), GTX5 (**G**), and GTX1/4 (**H**). Data points represent the mean ± SE from triplicate measurements.

Similarly, the potential inhibitors identified in the above preliminary screening, including dcGTX2&3, STX, GTX1/4, dcSTX, GTX2/3, and GTX6, were further tested by measuring their concentration–response curves through serial dilution to confirm their inhibitory effects on hNav1.1. The results (Figure 6) demonstrated that the fluorescence intensity, stimulated by 30 μM veratridine, significantly decreased in the presence of these compounds, further validating their inhibitory properties. The IC_50_ values for dcGTX2&3, STX, GTX1/4, dcSTX, GTX2/3, and GTX6 were 103.3 nM, 5.228 nM, 0.439 nM, 6.30 nM, 16.64 nM, and 86.28 nM, respectively. Consistent with the preliminary screening results (Figure 4B), STX, GTX1/4, dcSTX, and GTX2/3 exhibited inhibitory effects comparable to, or even stronger than, TTX (IC_50_ = 18.41 nM), while dcGTX2&3 and GTX6 showed relatively weaker inhibition. Additionally, GTX5 and C1&C2, which did not show inhibitory effects in the preliminary screening (Figure 4B), were able to completely suppress the fluorescence signal when their concentrations were increased to 3 μM (Figure 6C,G). Therefore, both of them are weak inhibitors of hNav1.1. STX, GTX, and C1&C2 share similar structures. These structural features may account for their similar binding sites on hNav1.1, yet differing inhibitory potencies [5].

**Figure 7:**
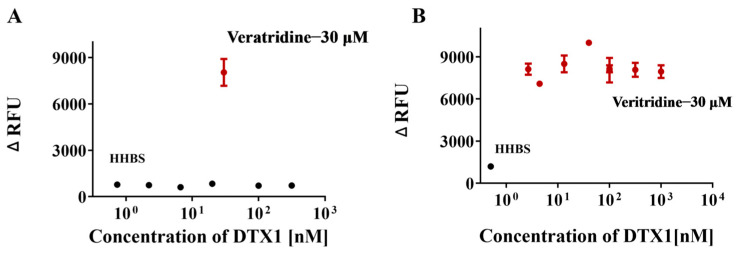
Concentration–response analysis of DTX1 in agonist mode (**A**) and inhibition mode (**B**). Data points represent the mean ± SE from triplicate measurements.

To further validate the specificity of this assay, we measured the fluorescence response of DTX1, a compound known not to interact with the hNav1.1 channel, in both agonist and inhibitor modes. In the preliminary screening mode (Figure 4), DTX1 did not exhibit any potential agonist or inhibitor activity. Further concentration–response curve analysis revealed that, in the agonist mode (Figure 7A), as the concentration of DTX1 increased, the fluorescence signal remained at baseline levels, with no significant increase even at a high concentration of 20 μM. In the inhibition mode (Figure 7B), as the concentration of DTX1 increased, the fluorescence intensity stimulated by 30 μM veratridine remained elevated and was not significantly inhibited. These observations suggest that DTX1 does not interact with the hNav1.1 channel, which is consistent with previous reports indicating that DTX1 is primarily associated with diarrhetic shellfish poisoning (DSP) rather than neurotoxicity [46], thus demonstrating the selectivity and specificity of the testing platform.

### 2.6. Validation of the Method by Comparison with the Patch-Clamp Technique

**Figure 8:**
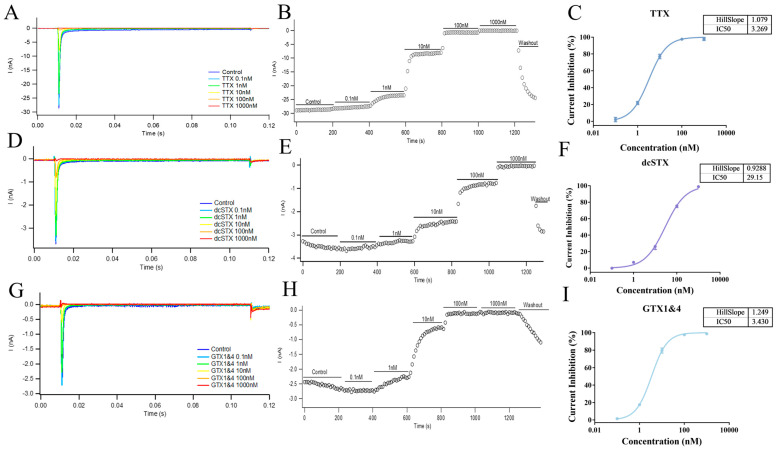
Effect of different concentrations (0.1 nM to 1000 nM) of TTX (**A**), dcSTX (**D**), and GTX1&4 (**G**) on hNav1.1 current signals measured by the patch-clamp technique. The currents were recorded from multiple experiments for TTX (**B**), dcSTX (**E**), and GTX1&4 (**H**), along with the resulting inhibition curves ((**C**): TTX, (**F**): dcSTX, (**I**): GTX1&4). The data points in (**C**,**F**,**I**) represent the mean ± SE from duplicate measurements in 2 cells.

The patch-clamp technique is an advanced electrophysiological technology that enables direct measurement and study of ion channel activity on cell membranes [47], and is often regarded as the gold standard for ion channel research. In this study, the whole-cell automated patch-clamp technique was used to validate the established fluorescence-based biosensing approach. Using the same hNav1.1-CHO cell line as in the fluorescence method, patch-clamp comparison was conducted with three representative compounds (TTX, dcSTX, and GTX1&4), measuring their effects on the hNav1.1 currents at different concentrations (ranging from 0.1 nM to 1000 nM). As shown in Figure 8A,D,G, with increasing concentrations of the three inhibitors, the peak current of hNav1.1-CHO cells significantly decreased. At concentrations of 100 nM, TTX and GTX1&4 almost completely suppressed the current signal, while dcSTX suppressed approximately 80% of the current (Figure 8B,E,H). Inhibition curves were plotted, and the IC_50_ values determined by patch clamp for TTX, dcSTX, and GTX1&4 were 3.269 nM, 29.15 nM, and 3.430 nM, respectively (Figure 8C,F,I), while the IC_50_ values determined by fluorescence were 18.41 nM, 6.302 nM, and 0.4389 nM, respectively. These results were in good agreement, confirming the strong inhibitory effects of the three compounds on hNav1.1 and further validating the reliability of the established fluorescence method.

## 3. Discussion

In this study, a high-throughput fluorescence assay based on hNav1.1-CHO cells was developed and validated against the patch-clamp technique, demonstrating reliable identification of both agonists and inhibitors of the hNav1.1 channel. The successful performance of this method relies on two essential improvements. First, the use of the hNav1.1-CHO cell line, as opposed to the commonly used HEK cell lines, provides enhanced stability and reproducibility, making it well suited for large-scale screening. More importantly, it significantly reduces interference from endogenous ion channels in the host cells, enabling the effective use of membrane potential-sensitive fluorescent dyes in this system.

Several fluorescence-based high-throughput assays have been developed for the discovery of Nav channel ligands. These methods measure intracellular sodium influx using sodium indicator dyes that emit fluorescence upon binding to sodium ions [48,49]. Commonly used sodium-specific fluorescence dyes include sodium-binding benzofuran isophthalate/acetoxymethyl ester (SBFI/AM), which enables passive diffusion across cell membranes [50,51,52,53]. However, these methods often require dual-wavelength excitation and additional steps, such as washing away extracellular dyes, which complicate the experimental procedure [54,55]. Membrane potential dyes, such as DiSBAC2(3) and FMP blue dye, have also been employed in high-throughput screening assays for Nav channel antagonists. However, these approaches either require the use of two agonists simultaneously [29] or are limited to inhibitor screening, showing minimal response to known agonists like veratridine and brevetoxin-2 [26]. This limitation may also be linked to the use of HEK cell lines in these methods. The Screen Quest™ Membrane Potential Assay Kit(AAT Bioquest, Pleasanton, CA, USA) enabled a homogeneous assay with a fast read time in our study. It employed a proprietary long-wavelength indicator to detect membrane potential changes caused by the opening and closing of sodium channels. The red fluorescence of the indicator increased upon entering cells, minimizing interference from tested compounds and cellular autofluorescence. With an excitation wavelength of 620 nm and an emission wavelength of 650 nm, the fluorescent dye translocated into the cells in response to a voltage-dependent change, separating from the extracellular quencher and resulting in fluorescence enhancement. Additionally, the optical response is highly sensitive, with a typical change of 1% of the fluorescence value per mV. The fluorescent indicator used in this method offers high sensitivity to membrane potential changes, minimal interference from extracellular dyes and cellular autofluorescence, distinct long-wavelength excitation and emission, rapid response times, and cost-effectiveness, making it ideal for detecting ion channel activity.

The high-throughput biosensing method established in this study was validated against the patch-clamp technique using three representative marine toxins, yielding satisfactory results. In our experiment, the IC_50_ of TTX derived from the inhibition curve was approximately 3.269 nM, which is consistent with the patch-clamp results reported by Tsukamoto (IC_50_ of TTX for Nav1.1 was 4.1 ± 0.2 nM, n = 4) [31]. They used the whole-cell patch-clamp technique to investigate the binding of 11 TTX analogues to Nav channel subtypes Nav1.1–Nav1.7. TTX was found to have a strong inhibitory effect on Nav channels, and the study identified the structural features responsible for the high-affinity binding of TTX and its analogues. Additionally, STX was another powerful tool for studying Nav channel activities, and its derivatives show promise as selective modulators of Nav channel subtypes. Shinohara [56] synthesized skeletal analogues of STX, FD-STX, as candidate Nav channel modulators, and evaluated their inhibitory effect on Nav1.4 and Nav1.5 channels using cell-based assays and the patch-clamp method. The IC_50_ values of (−)-FD-STX and (−)-FD-dcSTX were (7.3 ± 2.7) μM and (15 ± 5.4) μM, respectively, in a cell-based assay—significantly weaker than the inhibitory effects of STX (IC_50_ = 5.228 nM) and its derivative dcSTX (IC_50_ = 6.302 nM) on hNav1.1 observed in our study.

In addition to inhibitors, some agonists have also been characterized using the patch-clamp technique in previous studies. Shinohara [27] used automated patch-clamp electrophysiology to assess the functional modulation of recombinant hNav channels exposed to brevetoxin3 and brevenal. The results showed that Nav1.2, Nav1.4, Nav1.5, and Nav1.7 were sensitive to brevetoxin3, with EC_50_ values from 5.3 ± 1.8 pM to 4.1 ± 1.0 nM, significantly lower than the EC_50_ value of brevetoxin3 for hNav1.1 (453.9 nM) in our experiment. Patch-clamp techniques were also employed to examine the interactions between veratridine and Nav channel subtypes [57,58], revealing veratridine’s dual action on sodium currents. For Nav1.6, the peak amplitude of the sodium current was enhanced by veratridine (1–10 μM), while higher concentrations (30 μM) suppressed the peak current, but evoked sustained currents and tail currents [57]. A similar phenomenon was observed for Nav1.7, where veratridine exerted a dose-dependent inhibitory effect on the peak current (IC_50_ = 18.39 µM) and also elicited tail and sustained currents (EC_50_ = 9.53 µM) [58]. Another high-throughput fluorescent sodium influx assay yielded results consistent with our screening experiments, showing veratridine as an agonist for Nav1.1–Nav1.7, with EC_50_ values ranging from 10 to 29 µM [51]. While patch-clamp techniques detect sodium current changes in individual cells, providing millisecond-scale temporal resolution for detailed mechanistic insights, cell-based fluorescence methods monitor cumulative sodium ion concentrations or changes in membrane potential over a period of time. Therefore, both methods complement each other when characterizing agonists. It is important to note that while fluorescence-based assays are effective for initial screening of compound activity, they do not control membrane potential as successfully as patch-clamp techniques. The membrane potential and channel state (resting, activated, or inactivated) are crucial in modulating the effects of many compounds, including veratridine. Consequently, compounds that demonstrate activity in fluorescence-based assays may not produce the same effects in a patch-clamp test, potentially leading to false positives or negatives. However, the high-throughput biosensing approach developed in this study demonstrates good comparability with the patch-clamp technique. Given that the patch-clamp technique is time-consuming, requires complex equipment, demands highly skilled operators, and disrupts intracellular equilibrium [59,60], the method presented here provides a convenient, reliable, and cost-effective alternative for the rapid screening of both agonists and inhibitors targeting hNav1.1.

Twelve marine toxins with different types of effects on hNav1.1 were tested in this study, yielding satisfactory results and offering a new option for marine toxin detection. Current methods for detecting marine toxin primarily include toxicity-based assays, such as mouse bioassays [61], cytotoxicity analysis [62], and receptor-target binding analysis [63], as well as structure-based methods like enzyme-linked immunosorbent assay (ELISA) [64], liquid chromatography–mass spectrometry (LC-MS) [65], and aptamer-based methods [66]. While toxicity-based assays, such as mouse bioassays and cytotoxicity tests, reflect the overall toxic effects of compounds, they do not clarify the mechanism and have long experimental durations. Structure-based methods typically use chromatography–mass spectrometry or structure-specific recognition for qualitative and quantitative determination, but they do not provide information about the toxicity. The method presented here enables more accurate and specific screening of compounds targeting hNav1.1, with clear mechanisms, and allows for the comparison of toxicity potencies.

At least nine receptor binding sites on sodium ion channels have been identified, with marine toxins binding to distinct sites [67]. For example, TTX and STX (site 1) interact with the P loop at domains I, II, III, and IV of the α-subunit. Veratridine (site 2) binds to segment S6 of domain II, while brevetoxins (site 5) bind to segment S6 of domain I [67]. These findings suggest that the fluorescence-based biosensing method developed in this study was able to identify hNav1.1 inhibitors and agonists bound to distinct receptor sites on the α-subunit, positioning it as a promising platform for universal and rapid screening.

Future studies could optimize this assay by enhancing its sensitivity to low-affinity compounds and expanding the screening to include additional Nav channel subtypes, thereby improving its applicability. Moreover, integration of this method with high-throughput drug screening platforms could facilitate the identification of novel therapeutic agents targeting Nav channels for the treatment of a broader range of diseases, including pain, neurological, and cardiac disorders.

## 4. Materials and Methods

### 4.1. Chemicals and Reagents

HAM’S/F-12 medium and penicillin–streptomycin solution were obtained from Hyclone (Logan, UT, USA), while veratridine was sourced from APExBIO (Houston, TX, USA). Fetal bovine serum (FBS), 0.25% Trypsin-EDTA, and Hank’s Balanced Salt Solution with 20 mM HEPES Buffer (HHBS) were supplied by Gibco (New York, NY, USA). Hygromycin B was purchased from Amresco (Solon, OH, USA), and phosphate-buffered saline (PBS) from Takara (Shiga, Japan). Poly-L-lysine solution (PLL) and DMSO were obtained from Sigma (Saint Louis, MO, USA). The fluorescence membrane potential assay kit (Screen Quest Membrane Potential Assay Kit Red Fluorescence) was obtained from AAT Bioquest (Pleasanton, CA, USA). Zeocin and blasticidin were purchased by Solarbio (Beijing Solarbio Science & Technology Co., Ltd., Beijing, China). Dimethyl Sulfoxide (DMSO), Sodium Chloride (NaCl), KCl, Ethylene Glycol Bis(2-aminoethylether)-N,N,N′,N′-tetra acetic acid (EGTA), MgCl_2_·6H_2_O, D-Glucose and CaCl_2_·2H_2_O were both obtained from Sigma (Saint Louis, MO, USA). Tetrodotoxin (purity ≥ 99%) was provided by Zhejiang Kangte Biotechnology (Hangzhou, China). Brevetoxin 1, 2, and 3 (purity ≥ 95%) were sourced from Taiwan Algal Science (Taoyuan, China). GTX1/4, GTX 2/3, GTX 5, GTX 6, DTX 1, STX, dcGTX 2&3, dcSTX, and C1&C2 were purchased from Health Canada (Ottawa, ON, Canada). All experiments utilized ultrapure water.

### 4.2. hNav1.1-CHO Cell Culture

A CHO cell line stably expressing the hNav1.1 channel (hNav1.1-CHO; human Nav1.1 gene information: SCN1A, NM_001165963, CDS size 6030 bp) was developed by ICE Bioscience (Beijing, China) and utilized in this study. The hNav1.1-CHO cells were cultured in F-12 medium supplemented with 10% FBS, 1% penicillin–streptomycin solution, and 200 μg/mL hygromycin B. The cells were maintained at 37 °C in a 5% CO_2_ incubator (CCl-170B-8, ESCO, Singapore), with experimental procedures as follows. A CHO-K1 cell line (ICE Bioscience, Beijing, China) served as a cell line blank control to verify that the hNav1.1-CHO cells have normal background fluorescence.

The cells were removed from the liquid nitrogen and promptly placed in a 37 °C water bath (Thermo Scientific™ Precision™, Waltham, MA, USA), with gentle shaking of the cryovial. After being thoroughly thawed, the cell suspension was transferred into 5 mL of pre-warmed complete medium at 37 °C, and the cell aggregates were gently blown off using a pipette. Cells were then collected by centrifugation at 1000 rpm for 5 min at room temperature using a TD25M centrifuge (YIDA, Dongguan, China). After the removal of the supernatant, the cells were seeded into a 6 cm culture dish with a final volume of 5 mL. The cell morphology and density were assessed using an inverted microscope (BDS200, OPTEC, Chongqing, China).

For subculture, the medium was removed, and the cells were washed once with PBS. Next, 1 mL of 0.25% Trypsin-EDTA solution was added, and the dish was incubated at 37 °C for 0.5 min. Once the cells detached from the bottom, 5 mL of complete medium pre-warmed to 37 °C was added. The cell suspension was gently pipetted to dissociate aggregates and transferred to a sterile centrifuge tube. Cells were collected by centrifugation at 1000 rpm for 5 min at room temperature, then seeded into 6 cm culture dishes at a density of 2.5 × 10^5^ cells per dish for expansion or maintenance. The correct expression and physiological activity of Nav 1.1 were characterized as described in the Supplementary Materials (Figure S1).

Prior to analysis, 96-well plates were coated with poly-L-lysine (PLL) overnight to ensure cell adherence. Then, 5 × 10^4^ hNav1.1-CHO cells were added per well. The plates were incubated for approximately 18 h, allowing the cells to adhere and reach about 80% confluence before the detection proceeded.

### 4.3. Initial Screening of Agonists

Hank’s Balanced Salt Solution (HBSS) supplemented with 20 mM HEPES (HHBS) was prepared and utilized in the subsequent experiments. Component A (10× MP Sensor) from the fluorescence membrane potential assay kit was thawed and aliquoted into 1 mL volumes. Prior to the detection, 1 mL of Component A was mixed with 9 mL of HHBS, vortexed, and prepared as a fluorescent dye working solution.

The stock solutions of each test compound were diluted with HHBS to an appropriate single-point concentration (5× the final concentration, with a recommended range of 1~50 μM). The 96-well plate containing hNav1.1-CHO cells was removed from the incubator and equilibrated to room temperature. After the culture medium was discarded, 100 μL of HHBS and 100 μL of fluorescent dye working solution were added to each well. The plate was incubated at 37 °C for 30 min, followed by an additional 30 min at room temperature.

Fluorescence measurements were performed using a microplate reader (SYNERGY4, BioTek, USA) with the following settings: excitation at 620 ± 10 nm and emission at 665 ± 8 nm. Test compounds were sequentially added by introducing 50 μL of each solution (5× the final concentration) to individual wells. Fluorescence was recorded for 1 min at 300 ms intervals after each addition, and this process was repeated for all compounds. Negative (HHBS) and positive (30 μM veratridine) controls were included in each batch for comparison.

### 4.4. Initial Screening of Inhibitors

The stock solutions of each test compound were diluted with HHBS to an appropriate single-point concentration (12.5× the final concentration; recommended range: 1~50 μM). Similarly, the veratridine stock solution was diluted with HHBS to a 5× working concentration of 150 μM. The 96-well plate containing hNav1.1-CHO cells was equilibrated to room temperature. After the culture medium was discarded, 80 μL of HHBS and 100 μL of the fluorescent dye working solution were added to each well. The plate was incubated at 37 °C for 30 min, followed by 30 min at room temperature.

Fluorescence was measured using a microplate reader as described in Section 4.3. Sequential compound addition was carried out by adding 20 μL of the 12.5× test compound to individual wells, followed by a 1 min fluorescence measurement with 300 ms intervals. Afterward, 50 μL of the 5× veratridine working solution was added to the well, and fluorescence was recorded for another 1 min. This process was repeated for each test compound. A negative control (HHBS + 30 μM veratridine) and positive control (100 nM TTX + 30 μM veratridine) were included in each batch for comparison.

### 4.5. Agonist Activation Curve and EC_50_ Determination

For potential agonists identified in the initial screening, concentration–response curves were further utilized to confirm their specific agonistic effects on hNav1.1. The potency of agonists was approximately evaluated by their EC_50_ values. The stock solutions of the test compounds were serially diluted with HHBS to prepare working solutions across 6–8 gradient concentrations. All subsequent procedures were consistent with those described in Section 4.3 for the initial screening of agonists. During the assay, each concentration of the test compound was added sequentially to individual wells, and concentration–response curves were obtained. Nonlinear regression analysis was used to fit the curves and calculate the EC_50_ values.

### 4.6. Inhibition Curve and IC_50_ Determination

Similarly, for potential inhibitors identified in the initial screening, concentration–response curves were further used to confirm their specific inhibitory effects on hNav1.1, with IC_50_ values calculated to estimate their potency. The stock solutions of the test compounds were serially diluted with HHBS to prepare 6–8 gradient concentrations. The veratridine stock solution was diluted with HHBS to a 5× working concentration of 150 μM. All other procedures were consistent with the inhibitor screening protocol described in Section 4.4. During the assay, each concentration of the test compound was added sequentially to individual wells, and concentration–response curves were obtained. Nonlinear regression analysis was used to fit the curves and calculate the IC_50_ values.

### 4.7. Electrophysiology

Automated patch-clamp recordings were performed using the whole-cell patch-clamp technique at a holding potential at −120 mV. The external and internal solutions are formulated as follows: Extracellular solution: 140 mM NaCl, 3.5 mM KCl, 1 mM MgCl_2_·6H_2_O, 2 mM CaCl_2_·2H_2_O, 10 mM D-Glucose, 10 mM HEPES, 1.25 mM NaH_2_PO_4_·2H_2_O, and pH = 7.4 with NaOH; pipette solution: 50 mM CsCl, 10 mM NaCl, 10 mM HEPES, 60 mM CsF, 20 mM EGTA, and pH = 7.2 with CsOH. The voltage was depolarized to Vtest for 100 ms, with slight modifications based on the initial current-voltage (I–V) recording, and then returned to −120 mV. This procedure was repeated at 10 s intervals to assess the effect of the test compound on the peak amplitude of hNav1.1 current. Data were collected by an EPC 10 amplifier (HEKA, Lambrecht, Germany) and stored in PatchMaster software (v2.15).

The patch clamp was operated by pulling the glass pipette using a micropipette puller. The micropipette filled with intracellular solution was loaded into the pipette holder and a coverslip lined with cells were placed in a recording chamber under an inverted microscope, then a micromanipulator was manipulated under an inverted microscope so that the pipette descended into the recording solution and the resistance was recorded (R_pi_^p^). After touching the cell, slight suction was applied to achieve a high-resistance seal (in the GΩ range). Fast capacitance compensation was performed and negative pressure was maintained to break the membrane into whole-cell configuration. Then, slow capacitance was compensated and experimental parameters such as series resistance (R_s_) were recorded. No leak subtraction occurred.

Drug delivery was initiated when cells were perfused with control extracellular solution until the current amplitude stabilized, and the next drug concentration was tested after the current reached an equilibrium block (which took approximately 5 minutes). The control and test solutions flew sequentially through the chamber from low to high concentration via a gravity-fed solution delivery system. During the experiment, the solutions were withdrawn from the chamber by a peristaltic pump. All tests were performed at room temperature.

For each recording, the current response to the compound (peak current compound) were normalized to blank control (peak current control) and the inhibition rate was calculated as follows:

Inhibition% = (1 − Peak current compound/Peak current control) × 100%(1)

the mean, standard deviation (SD) and standard error (SE) were calculated for each test group, and data were presented as the mean ± SE.

The IC_50_ value was determined by fitting the concentration–response curve using the following nonlinear regression equation:

(2) $$Y=\mathrm{Bottom}+(\mathrm{Top}-\mathrm{Bottom})/(1+10^{(LogIC50-LogX)\times HillSlope})$$

the IC_50_ represents the half maximal inhibitory concentration.

## 5. Conclusions

In this study, we developed a novel high-throughput fluorescence-based biosensing method for the rapid screening of compounds targeting the hNav1.1 channel, utilizing a stable hNav1.1-CHO cell expression system. This method offers significant improvements, including high stability, reproducibility, minimal interference from endogenous ion channels, and the ability to screen both agonists and inhibitors. It also allows for the identification of compounds interacting with distinct binding sites on hNav1.1, providing both qualitative and semi-quantitative information on their binding affinities. Notably, the method was validated through comparison with the gold standard patch-clamp technique and successfully tested with 12 marine toxins, yielding consistent results. Moreover, its high-throughput, cost-effective, and rapid nature makes it an ideal alternative for large-scale and early-stage drug discovery and toxin screening.

## Data Availability

All data obtained during this study are available from the corresponding author on reasonable request.

## Supplementary Materials

The following supporting information can be downloaded at https://www.mdpi.com/article/10.3390/md23030119/s1, Figure S1: Expression of Nav1.1 mRNA in the stable cell line (A) and unadministered Nav1.1 current diagram (B).

## Abbreviations

The following abbreviations are used in this manuscript:

Nav	Voltage-gated sodium.
hNav1.1	Human Nav1.1.
hNav1.1-CHO	CHO cells stably expressing hNav1.1 α-subunit.
VSD	Voltage-sensing domain.
TTX	Tetrodotoxin.
STX	Saxitoxin.
HEK	Human Embryonic Kidney.
CHO	Chinese Hamster Ovary.
GTX1/4	Gonyautoxin ¼.
dcGTX 2&3	Decarbamoylgonyautoxin 2&3.
dcSTX	Decarbamoylsaxitoxin.
C1&C2	N-sulfocarbamoylgonyautoxin 2 and 3.
DTX1	Dinophysistoxin 1.
DSP	Diarrhetic shellfish poisoning.
SBFI/AM	Sodium-binding BenzoFuran Isophthalate/AcetoxyMethyl Ester.
ELISA	Enzyme-linked immunosorbent assay.
LC-MS	Liquid chromatography–mass spectrometry.
FBS	Fetal bovine serum.
HBSS	Hank’s Balanced Salt Solution.
HHBS	HBSS supplemented with 20 mM HEPES.
PBS	Phosphate-buffered saline.
PLL	Poly-L-lysine.
DMSO	Dimethyl sulfoxide.
EGTA	Ethylene Glycol Bis(2-aminoethylether)-N,N,N′,N′-tetra acetic acid.
