# Supplementary material for: A High-Throughput Biosensing Approach for Rapid Screening of Compounds Targeting the hNav1.1 Channel: Marine Toxins as a Case Study
Source: Mar Drugs. 2025 Mar 9;23(3):119. doi: 10.3390/md23030119 (PMC11943507; doi:10.3390/md23030119)
Supplement: Supplementary file 1 [file marinedrugs-23-00119-s001.zip › marinedrugs-3488318-supplementary.pdf]

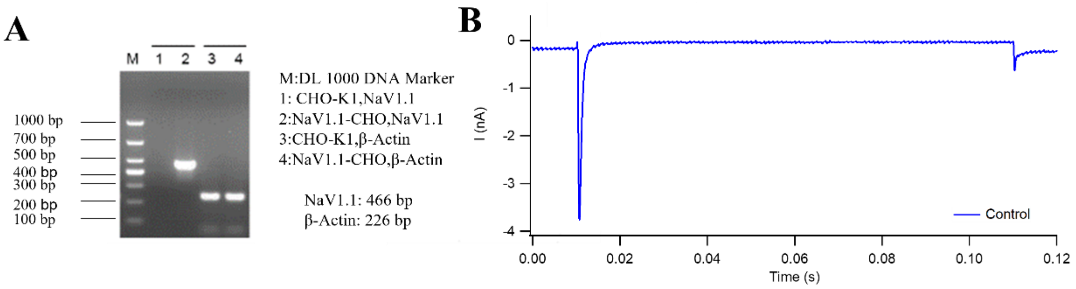

Figure S1. Expression of Nav1.1 mRNA in the stable cell line (A) and hNav1.1-CHO current diagram (B).

The gel electrophoresis results clearly show the expected PCR product for Nav1.1 in the hNav1.1-CHO cells. As indicated in figure S1 A, a clear band at 466 bp confirmed the successful amplification of the Nav1.1 gene, validating its expression in the transfected cells (Lane 2). Moreover, CHO-K1 cells without Nav1.1 transfection (Lane 1) served as a negative control. As expected, no band was observed at the 466 bp position, which supported the absence of Nav1.1 expression in the CHO-K1 cells. Additionally, the β-Actin gene was successfully amplified, as shown by the 226 bp band in both the CHO-K1 and hNav1.1-CHO cells.

Figure S1B shows the patch-clamp current signal of the hNav1.1 channel in the absence of any external substance, demonstrating that the hNav1.1-CHO cell line retains normal electrophysiological function.
